# Lentiviral-based anti-HIV therapeutic vaccine: design, preclinical studies and phaseI/II clinical trial preliminary results

**DOI:** 10.1186/1471-2334-14-S2-P81

**Published:** 2014-05-23

**Authors:** Cécile Bauche, Marie Rodriguez, Emeline Sarry, Ana Bejanariu, Emmanuelle Sabbah-Petrover

**Affiliations:** 1 THERAVECTYS, Villejuif, France

## 

THERAVECTYS, a spin off the Pasteur Institute, develops a new generation of prophylactic and therapeutic vaccines using optimized lentiviral vectors. It’s most advanced product, a therapeutic anti-HIV vaccine treatment, has entered clinical Phase I/II end of 2012. This vaccination should allow seropositive patients to gain an immunological status identical to the so-called “Functional Cured” patients who develop an efficient immunological response capable of controlling the infection without therapy.

Vaccine candidates are integrative and self-inactivated live-recombinant lentiviral vectors. They encode an HIV antigen, under the regulation of a patented promoter that is preferentially induced in APC (generating the specific immune response), and showing a basal level expression in all cells (allowing their elimination by the settled immune response).These vaccine candidates are classified as “Live recombinant vectored vaccines” (EMA, 2011).

Preclinical studies demonstrated i)the generation of a strong, specific and very long lasting T-cell immune response (up to 2 years in murine animal models), ii) the restricted diffusion of the vaccine candidates after injection and iii)their fast disappearance within few weeks, correlated with an absence of macroscopic and microscopic toxicity.

These data allowed the settlement of the anti-HIV therapeutic Phase I/II clinical trial that is held in France and Belgium and that has ended the enrollment of the 36 HIV-1 infected patients. THERAVECTYS’ anti-HIV vaccine treatment is assessed at three doses and safety, tolerability and immunogenicity compared to a placebo group. Furthermore, vaccine efficiency is be evaluated by the interruption of the HAART treatment in all patients, including placebo. Final results are expected by 2014 with intermediary analysis in April 2014.

